# Postprandial Glycemia, Insulinemia, and Antioxidant Status in Healthy Subjects after Ingestion of Bread made from Anthocyanin-Rich Riceberry Rice

**DOI:** 10.3390/nu12030782

**Published:** 2020-03-16

**Authors:** Charoonsri Chusak, Porntip Pasukamonset, Praew Chantarasinlapin, Sirichai Adisakwattana

**Affiliations:** 1 Phytochemical and Functional Food Research Unit for Clinical Nutrition, Department of Nutrition and Dietetics, Faculty of Allied Health Sciences, Chulalongkorn University, Bangkok 10330, Thailand; Charoonsri.c@gmail.com (C.C.); chantarasinlapin.p@gmail.com (P.C.); 2 Addlife Anti-Aging Center, Q House Lumpini Building, 1 South-Sathon Rd., Tungmahamek, Sathorn, Bangkok 10120, Thailand; Pasukamonset@gmail.com

**Keywords:** Riceberry rice, bread, postprandial, glycemic response, antioxidant status

## Abstract

Riceberry rice, a gluten-free grain, contains many nutrient components, including carbohydrates, proteins, certain fatty acids, and micronutrients, as well as bioactive non-nutrient compounds, such as polyphenolic compounds. This study aimed to evaluate the effect of bread made from anthocyanin-rich Riceberry rice on the postprandial glycemic response, glucagon-like peptide-1 (GLP-1), antioxidant status, and subjective ratings of appetite. In the crossover design, 16 healthy participants (six men and 10 women) completed four sessions involving blood collection in the fasting state and at 30, 60, 90, 120, 150, and 180 min after food consumption (50 g of available carbohydrate) in a randomized order: 1) glucose solution, 2) wheat bread (WB), 3) Riceberry rice bread (RRB), and 4) Hom Mali bread (HMB). Consumption of RRB resulted in significantly lower postprandial plasma glucose concentration at 30 and 60 min when compared to HMB. No difference in postprandial glucose concentration between RRB and WB was observed. In addition, postprandial plasma insulin showed a significant decrease in the group which received RRB at 15 and 60 min, as compared to HMB. In comparison with 50 g of glucose, as a reference, the glycemic index (GI) of RRB, WB, and HMB was 69.3 ± 4.4, 77.8 ± 4.6, and 130.6 ± 7.9, respectively. Interestingly, the ferric-reducing ability of plasma (FRAP) level was shown to significantly increase after consumption of RRB. In the meantime, a significant decrease in the postprandial FRAP level was also observed following an intake of WB and HMB. All breads caused increases in the postprandial plasma protein thiol group and had similar effects on hunger, fullness, desire to eat, and satiety ratings. However, consumption of RRB, WB, and HMB did not change plasma GLP-1 and malondialdehyde (MDA) levels when compared to the baseline. The findings suggest that anthocyanin-rich Riceberry rice can be a natural ingredient for gluten-free bread which reduced glycemic response together with improvement of antioxidant status in healthy subjects.

## 1. Introduction

Polyphenols are the most diverse group of phytochemical compounds distributed in vegetables and fruits. Today, there is an increasing interest in the biological activity of polyphenols because of their beneficial effects on human health. A meta-analysis of epidemiological studies revealed that long-term consumption of a plant-based diet rich in polyphenols has been associated with reduction in the risk of major non-communicable chronic diseases, such as diabetes [1] and cardiovascular diseases [2]. Several published reports have amassed supporting recommendations to consume plants containing phytochemical compounds for reduction of carbohydrate digestion and monosaccharide absorption [3,4]. One of the possible mechanisms to reduce carbohydrate digestion is the limitation of activity of digestive enzymes, including α-amylase and α-glucosidase [3]. Besides, bioactive compounds in natural plants also display several biological activities, such as antioxidant and anti-inflammatory effects [5]. Anthocyanins, the common class of plant-based polyphenol, are responsible for the red, purple, and blue color in fruits and vegetables. Previous studies demonstrated that ingestion of anthocyanins strawberry resulted in a reduction in postprandial glucose, insulin, and inflammatory responses and increased postprandial antioxidant status in overweight people who consumed a high-carbohydrate and moderate-fat meal [6]. Interestingly, consumption of blueberry is associated with a diet-induced increase in postprandial antioxidant status in the subjects [7,8]. Moreover, a study of Jokioja et al. demonstrated that consumption of anthocyanin-enriched purple potato reduced postprandial glycemia and insulinemia in healthy male volunteers when compared to the group who consumed yellow potato [9]. Chusak et al. reported that acute ingestion of an anthocyanin-rich *Clitoria ternatea* beverage attenuated postprandial glucose together with improvement of plasma antioxidant capacity when consumed with sucrose [10]. In this regard, plant-based anthocyanins may be considered as a natural ingredient for suppression of postprandial glucose and protection of postprandial oxidative stress.

Rice, a staple food in many parts of the world, has been recognized as an excellent source of gluten-free ingredients [11]. However, it is also rich in carbohydrates and may provide more food energy than any other type of plant. Recently, Riceberry rice, a deep-purple grain, was developed by a cross-bred strain between Hom Nin rice (known as a high-antioxidant rice) and Hom Mali 105 rice (known as a fragrant rice). This rice contains high gamma-oryzanol, β-carotene, niacin, thiamin, vitamin B2, and total phenolic compounds, especially anthocyanins [12]. The major anthocyanin identified in the pigment of Riceberry rice was cyanidin-3-*O*-glucoside and peonidin-3-*O*-glucoside [13]. Riceberry rice extract has been previously reported as possessing several biological properties, such as antioxidant, anti-advanced glycation-end products (AGEs), anti-inflammatory, and hepatoprotective activities [14,15]. Recent research provides important evidence that the pigment of Riceberry rice influences the key step of carbohydrate digestion and absorption by inhibiting intestinal α-glucosidase and suppressing mRNA expression of sodium-dependent glucose cotransporter 1 (SGLT1) [13]. Interestingly, Riceberry rice can be successfully applied for the development of gluten-free products, such as rice-based low-glycemic dysphagia diets. This product has been used as a special eating plan for people who have moderate to severe trouble swallowing [16].

Interestingly, Riceberry rice has been used to produce commercial gluten-free rice flour and associated food products. Thiranusornkij et al. (2019) discovered that Riceberry rice flour had higher levels of total polyphenols, anthocyanins, and ferric-reducing activity power (FRAP) than Hom Mali flour. In addition, bread made from Riceberry rice flour has been found to contain a higher proportion of undigested starch, leading to a decreased rate of starch hydrolysis [17]. Despite data available from in vitro experiments, there is very limited information regarding the impacts of gluten-free Riceberry rice bread on human health after consumption. Therefore, the aim of this study was to investigate the acute effect of single administration of breads made from anthocyanin-rich Riceberry rice (RRB) on postprandial glycemia, insulinemia, glucagon-like peptide-1 (GLP-1), antioxidant status, and the subjective rating of appetite in healthy subjects. To compare the effectiveness of RRB, the effect of consumption of breads made from Hom Mali rice (HMB) and wheat (WB) was also determined.

## 2. Materials and Methods

### 2.1. Sample Preparation

**Figure 1:**
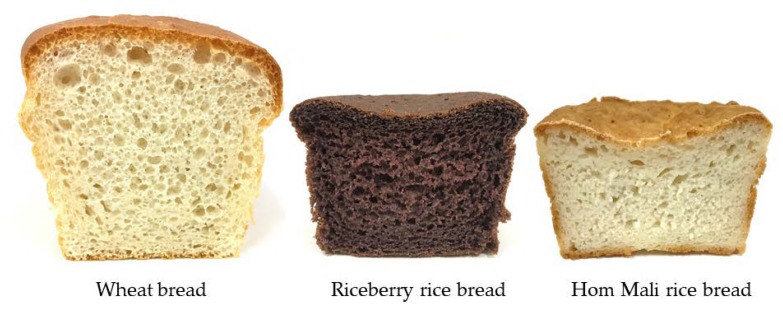
The cross-sections of wheat bread (WB), Riceberry rice bread (RRB), and Hom Mali rice bread (HMB), respectively.

Riceberry rice and Hom Mali rice were ground and sieved using 150 meshes and made into powder, respectively. The rice powder was stored in a sealed bag at room temperature until use. In addition, the study bread was prepared by KCG Corporation Co. Ltd. using the procedure of Chusak et al. [18]. The RRB and HMB were prepared by replacing wheat flour with 100% (*w*/*w*) of Riceberry rice or Hom Mali rice flour, respectively. All breads were prepared the day before the study. Cross-sections of bread made from wheat flour, Riceberry rice, and Hom Mali rice powder are shown in Figure 1. Proximate analysis of the bread samples and total dietary fiber were carried out using the Association of Official Analytical Chemists (AOAC) method [19].

### 2.2. Participants

Healthy young male and female volunteers (*n* = 20) were enrolled in this trial through advertisements on the university bulletin board and social media. The recruitment of the participants was performed by a researcher at the Faculty of Allied Health Sciences, Chulalongkorn University. Measurements of fasting blood glucose, total cholesterol, triglyceride, and LDL cholesterol were conducted by the Health Sciences Service Unit, Faculty of Allied Health Sciences, Chulalongkorn University. The participants performed a self-administered questionnaire containing the inclusion and exclusion criteria. Inclusion criteria included: age of 20–40 years, BMI 18.5–22.9 kg/m^2^, a fasting blood glucose concentration of <100 mg/dL, a fasting total cholesterol concentration of <200 mg/dL, a fasting triglyceride concentration of <150 mg/dL, a fasting LDL cholesterol concentration of <130 mg/dL, and being free of serious illness. The exclusion criteria included: known history or presence of gastrointestinal disease, metabolic diseases or type 1 and type 2 diabetes, pregnancy or lactating, smoking, alcohol abuse, known food allergies, intolerance or gastrointestinal problems to wheat, gluten or starchy foods, and taking supplementations or medications known to influence glucose tolerance or gastric emptying. Finally, 16 healthy participants (six men and 10 women) who met these criteria were invited to the laboratory and involved in duplicated oral glucose tolerance tests (OGTTs). The informed consent forms were signed by 16 eligible individuals to participate in the study. This study protocol was approved by the office of Ethics Review Committee for Research Involving Human Research Subjects, Human Science Group, Chulalongkorn University (COA No. 270/2561). The trial was registered with the Thai Clinical Trials Registry (TCTR20190113001) and was conducted between February and July 2019.

### 2.3. Study Design

The study was carried out using a randomized and crossover design with a 1-week wash-out period. Participants were randomly assigned to white wheat bread (WB), Riceberry rice bread (RRB), or Hom Mali 105 rice bread (HMB) by using a computer to generate random numbers with simple randomization. The study investigator generated the randomization plan, then assigned participants to the study treatments. The foods were tested once, and the glucose reference beverages (50 g glucose) were each tested twice.

Participants were asked to consume 250 mL of a drink containing 50 g of glucose and then randomly assigned to consume three types of bread: WB, RRB, and HMB containing 50 g of available carbohydrate (Table 1), respectively. To calculate the available carbohydrate, the amount of dietary fiber was analyzed and subtracted from the total carbohydrate. The order of the breads was randomly allocated to each participant through the use of a Latin square randomization sequence by the study investigator. The participants were instructed to avoid polyphenol-containing foods for at least 3 days prior to the study. They were also advised to maintain their lifestyle habits throughout the study. In addition, they were asked to avoid alcohol intake and heavy exercise for 1 day before each experimental test.

**Figure 2:**
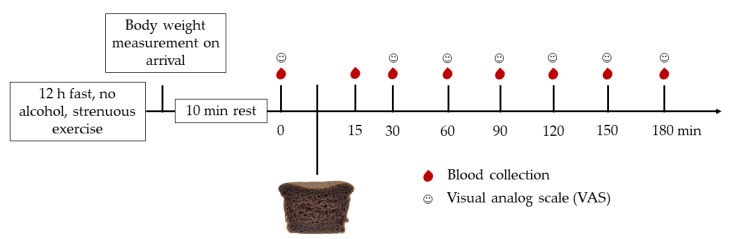
Study visit protocol.

After overnight fasting, participants arrived at the Faculty of Allied Health Sciences, Chulalongkorn University before 8:00 a.m. on the test day. The intravenous catheter was inserted into an antecubital vein, and a fasting blood sample withdrawn by the registered nurse. Then, the participants were instructed to consume the study food within 10–15 min. The time when the participants stared to ingest the study food was set as 0 min. Further blood samples were obtained before (0 min) and at 15, 30, 60, 90, 120, 150, and 180 min after starting consumption of each study food. Moreover, the subjective ratings of hunger, fullness, desire to eat, and satiety were evaluated by the visual analog scale (VAS) rating using a 100 mm scale ranging from 0 (“not at all”) to 100 (“extremely”). The scores were recorded at the time of blood collection (Figure 2). A maximum of 500 mL of water was prepared for the participants during the study. After the last blood sample collection, all participants were offered a meal.

### 2.4. Biochemical Measures

Plasma was obtained from EDTA and NaF tubes before centrifugation at 3000 rpm for 10 min at 4 °C. The plasma samples were taken and kept at −20 °C until analysis. Plasma glucose, insulin, and GLP-1 concentration were measured by using the glucose oxidase method (HUMAN GmbH, Germany) and Human Insulin and GLP-1 ELISA kit (Biobase Meihua Trading Co., Ltd., China), respectively.

### 2.5. Antioxidant and Oxidant Measures

The FRAP level was measured according to a previously published report [20]. The plasma sample (10 μL) was mixed with FRAP reagent (90 μL) containing 0.3 M sodium acetate buffer (pH 3.6), 10 mM 2,4,6-tri(2-pyridyl)- 1,3,5-triazine (TPTZ) in 40 mM HCl, and 20 mM FeCl_3_ and then incubated in the dark at room temperature for 30 min. The absorbance was measured at 595 nm. The FRAP level was expressed as FeSO_4_ equivalents. Moreover, the level of plasma thiol group was measured by an Ellman’s assay [21] with a slight modification. The plasma sample (90 μL) was mixed with 2.5 mM 5,5′-dithiobis-(2-nitrobenzoic acid) (DTNB) in 0.1 M PBS, pH 7.4 and then incubated at room temperature for 15 min. The absorbance was read at 410 nm. The plasma thiol level was expressed as μM L-cysteine equivalent. The level of plasma malondialdehyde (MDA) was determined according to a previously described report [10]. Plasma MDA was quantified using a method based on the formation of thiobarbituric acid reactive substances (TBARS). The plasma sample (150 μL) was mixed with 10% trichloroacetic acid (TCA) and 50 mM 2,6-di-tert-butyl-4-methylphenol (BHT). The mixture was centrifugated at 13000 rpm at 4 °C for 10 min and 0.67% thiobarbituric acid (TBA) was further added to the supernatant. After heating at 95 °C for 10 min, the absorbance was measured at 532 nm. The plasma MDA level was reported as an mM MDA equivalent.

### 2.6. Statistical Analysis

The sample size for this study was calculated and based on a previous study of postprandial responses that reported a significant reduction in glucose [22]. Therefore, the sample size was determined by designing the trial to have a 95% confidence level and 80% power of test in order to detect a significant difference in glucose with a minimum of 13 participants. Accounting for a drop-out rate of 20%, 16 participants were recruited for the study.

Data were presented as means ± SEM. All statistical analyses were performed with SPSS software (version 22.0, IBM Corp., Armonk, NY, USA) with *p* < 0.05 considered to be significant. Data were reported as changes from baseline and compared by a repeated measures ANOVA and Duncan’s post-hoc test. The incremental area under the curves (iAUCs) of glucose, insulin, GLP-1, FRAP, MDA, and thiol were calculated by the trapezoidal rule. The iAUCs were statistically performed using one-way ANOVA followed by Duncan’s post-hoc test. The glycemic index (GI) of WB, RRB, and HMB was determined by the mean individual ratios resulting from iAUCs for glucose over 120 min after consumption of each bread and ingestion of the glucose solution multiplied by 100.

## 3. Results

### 3.1. Participants

**Figure 3:**
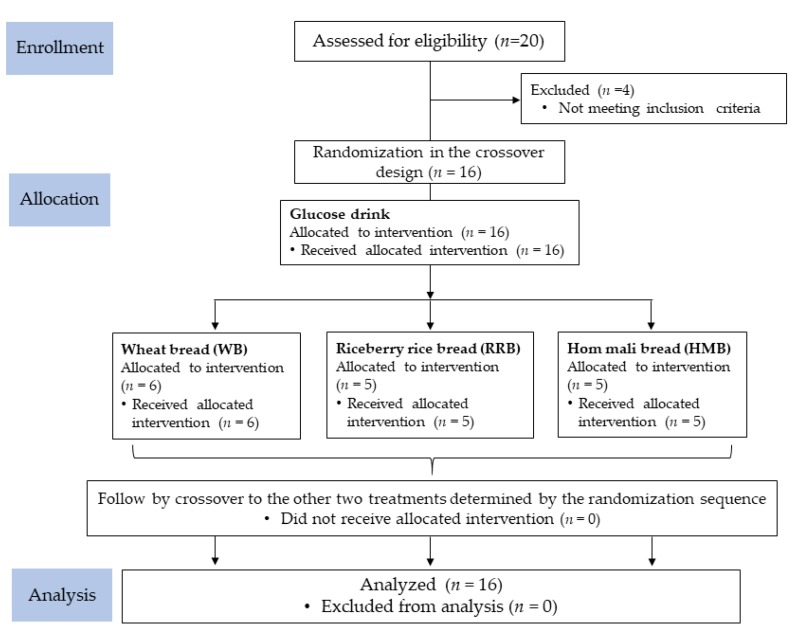
Consolidated Standards of Reporting Trials (CONSORT) diagram for the study trial.

The baseline characteristics of the participants are described in Table 2. Twenty participants were assessed for eligibility, where only 16 were randomly assigned to the test food order, as shown in Figure 3. Four participants were excluded from the study as they did not meet the criteria. A total of 16 participants completed all test interventions.

### 3.2. Postprandial Plasma Glucose

**Figure 4:**
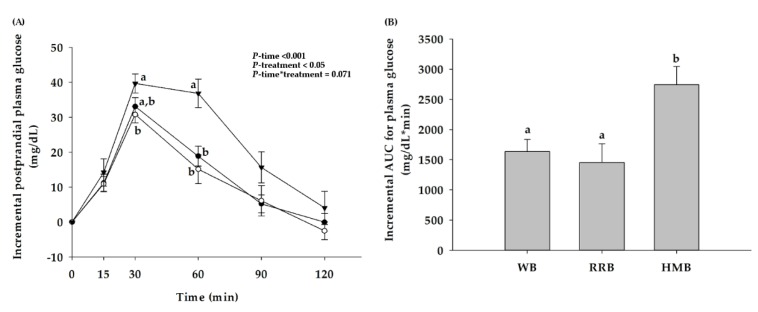
(**A**) Changes in postprandial glucose concentration and (**B**) the incremental area under the curves (iAUCs) for plasma glucose of healthy participants after the consumption of wheat bread (WB), Riceberry rice bread (RRB), and Hom Mali bread (HMB). All values are means ± SEM, *n* = 16. WB (●), RRB (○), and HMB (▼). For each time point, means with a different letter are significantly different, *p* < 0.05.

Postprandial plasma glucose changes after ingestion of WB, RRB, and HMB are shown in Figure 4A. When comparing with the baseline, consumption of WB, RRB, and HMB showed a significant increase in postprandial glucose concentration after 15, 30, and 60 min. The peak concentration of postprandial glucose of all groups was reached at 30 min. Moreover, there were significant differences in postprandial glucose between RRB, HMB, and WB. Consumption of RRB resulted in a significantly lower postprandial plasma glucose concentration at 30 and 60 min when compared to HMB. In the meantime, an intake of WB showed lower postprandial glucose at 60 min than that of HMB. However, no significant difference in postprandial glucose concentration between RRB and WB was noticed throughout the 120 min.

The iAUCs for postprandial plasma glucose of breads are illustrated in Figure 4B. RRB and WB resulted in a 60% and 53% lower glycemic AUC response than HMB, respectively. In comparison with the iAUC of glucose drink, the glycemic index (GI) of RRB, WB, and HMB was 69.3 ± 4.4, 77.8 ± 4.6, and 130.6 ± 7.9, respectively.

### 3.3. Postprandial Plasma Insulin

**Figure 5:**
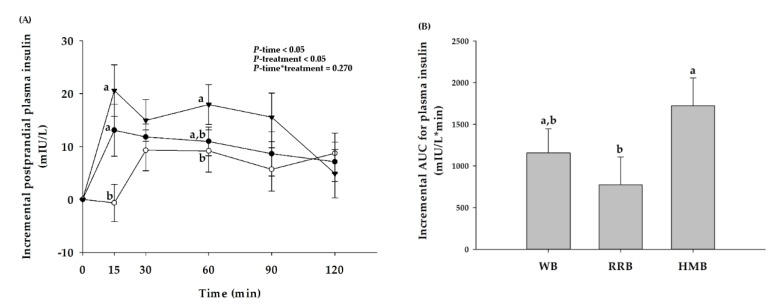
(**A**) Changes in postprandial insulin concentration and (**B**) the iAUCs for plasma insulin of healthy participants after the consumption of wheat bread (WB), Riceberry rice bread (RRB), and Hom Mali bread (HMB). All values are means ± SEM, *n* = 16. WB (●), RRB (○), and HMB (▼). For each time point, means with a different letter are significantly different, *p* < 0.05.

Plasma insulin responses after consumption of WB, RRB, and HMB are presented in Figure 5A. Ingestion of WB and HMB increased postprandial plasma insulin at 15 and 30 min. In contrast, consumption of RRB significantly attenuated the elevation of postprandial insulin concentration throughout the 120 min of the experiment. At 15 min of ingestion, the level of postprandial plasma insulin of the group that received RRB was lower than that of HMB and WB. The iAUCs for postprandial plasma insulin of breads are shown in Figure 5B. When compared to HMB, a 2.2-fold reduction of iAUCs for postprandial plasma insulin was detected after consumption of RRB. The iAUC of WB was similar to the values of RRB and HMB.

### 3.4. Plasma GLP-1

**Figure 6:**
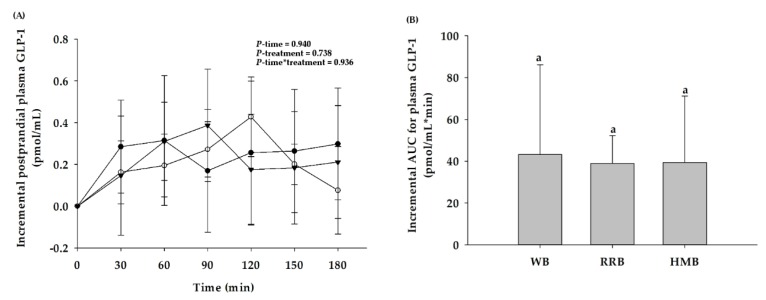
(**A**) Postprandial plasma GLP-1 changes and (**B**) the iAUCs for plasma GLP-1 in healthy participants after the consumption of wheat bread (WB), Riceberry rice bread (RRB), and Hom Mali bread (HMB). All values are means ± SEM, *n* = 16. WB (●), RRB (○), and HMB (▼). For each time point, means with a different letter are significantly different, *p* < 0.05.

In comparison to the baseline, consumption of WB, RRB, and HMB had no effect on an increase in postprandial GLP-1. Furthermore, there were no significant differences in postprandial plasma GLP-1 levels and iAUCs of the test breads (Figure 6A,B).

### 3.5. Plasma Antioxidant Status

**Figure 7:**
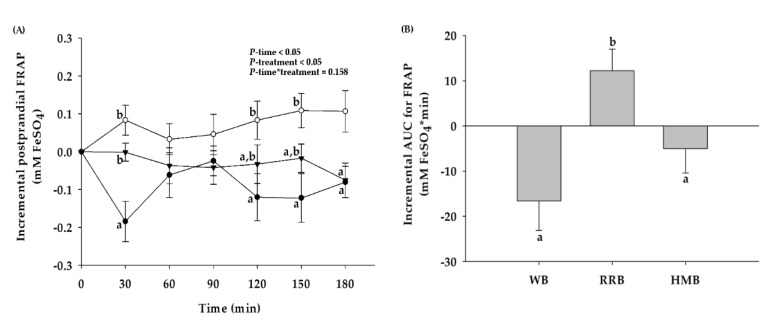
(**A**) Changes in postprandial ferric-reducing activity power (FRAP) level and (**B**) the iAUCs for FRAP level of healthy participants after the consumption of wheat bread (WB), Riceberry rice bread (RRB), and Hom Mali bread (HMB). All values are means ± SEM, *n* = 16. WB (●), RRB (○), and HMB (▼). For each time point, means with a different letter are significantly different, *p* < 0.05.

When compared to the baseline, consumption of WB and HMB markedly decreased the FRAP level (Figure 7A). Interestingly, RRB resulted in an increase in FRAP level at 30, 120, 150, and 180 min. The iAUC for postprandial plasma FRAP after RRB consumption was greater than that of WB and HMB, respectively (Figure 7B).

**Figure 8:**
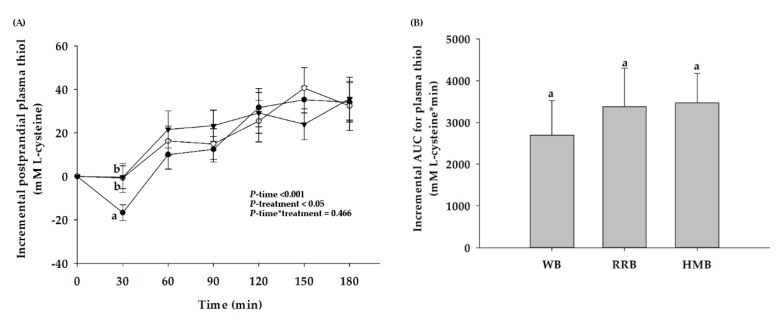
(**A**) Changes in postprandial thiol level and (**B**) the iAUCs for plasma thiol level of healthy participants after the consumption of wheat bread (WB), Riceberry rice bread (RRB), and Hom Mali bread (HMB). All values are means ± SEM, *n* = 16. WB (●), RRB (○), and HMB (▼). For each time point, means with a different letter are significantly different, *p* < 0.05.

Consumption of RRB and HMB did not change plasma thiol concentration from the baseline at 30 min, whereas a decrease in plasma thiol was markedly seen in the group which received WB (Figure 8A). After that, three breads significantly increased postprandial plasma thiol concentration after 120 min ingestion. However, the iAUCs for postprandial plasma thiol did not differ among the tested breads (Figure 8B).

**Figure 9:**
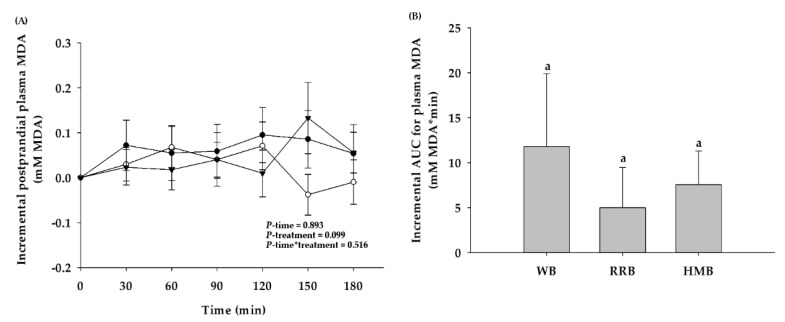
(**A**) Changes in postprandial malondialdehyde (MDA) level and (**B**) the iAUCs for plasma MDA level of healthy participants after the consumption of wheat bread (WB), Riceberry rice bread (RRB), and Hom Mali bread (HMB). All values are means ± SEM, *n* = 16. WB (●), RRB, (○), and HMB (▼). For each time point, means with a different letter are significantly different, *p* < 0.05.

According to the generation of lipid peroxidation, the levels of postprandial plasma MDA after ingestion of all breads are presented in Figure 9A. There were no changes in postprandial plasma MDA after consumption of WB, RRB, and HMB, as compared to the baseline. Furthermore, the iAUCs of all breads were similar (Figure 9B).

### 3.6. Subjective Rating of Hunger, Fullness, Desire to Eat, and Satiety

**Figure 10:**
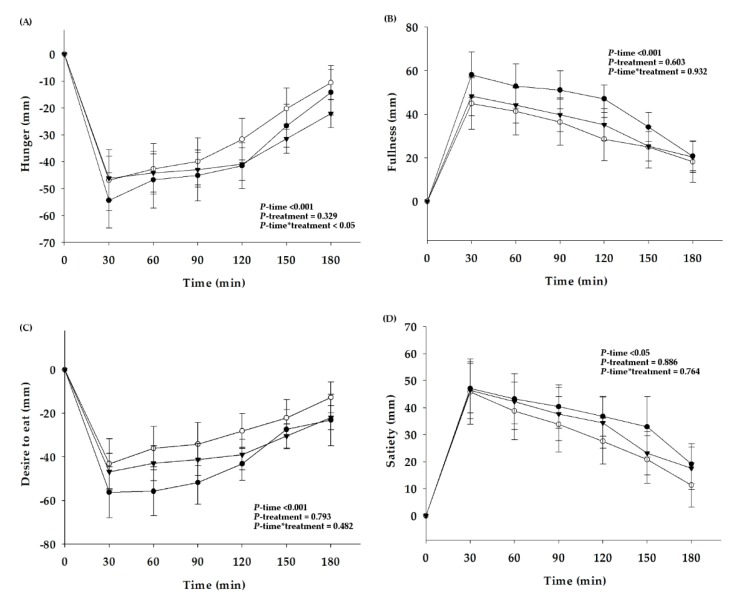
(**A**) Changes in hunger, (**B**) fullness, (**C**) desire to eat, and (**D**) satiety of healthy participants after the consumption of wheat bread (WB), Riceberry rice bread (RRB), and Hom Mali bread (HMB). All values are means ± SEM, *n* = 16. WB (●), RRB, (○), and HMB (▼). For each time point, means with a different letter are significantly different, *p* < 0.05.

As demonstrated in Figure 10A–D, consumption of WB, RRB, and HMB caused significant alteration of the hunger, desire to eat, fullness, and satiety ratings when compared to the baseline. Furthermore, there were no significant differences in the ratings of hunger, fullness, desire to eat, and satiety among the tested breads. The ratings of hunger, desire to eat, fullness, and satiety had similar scores following consumption of the three tested breads.

## 4. Discussion

Carbohydrates are the most important nutrient to modulate and influence postprandial glucose and insulin secretion in the blood circulation [23]. This is the first study to investigate the effect of RRB on postprandial glycemic, insulin response, antioxidant capacity, and satiety in healthy subjects. Our study found that all breads containing 50 g of available carbohydrate promoted a rise in the postprandial blood glucose peak at 30 min, which gradually decreased to the baseline after 120 min consumption. The results showed no significant difference between postprandial glucose and insulin obtained from WB and RRB consumption. When compared with HMB, RRB showed a significantly lower peak and postprandial plasma glucose and insulin concentration. This phenomenon may be attributed to the starch components of RRB and HMB [13]. Previous research revealed that anthocyanin-rich Riceberry rice bread had a higher level of undigestible starch than Hom Mali rice bread [17]. Ingestion of foods containing undigestible starch is clearly linked to a lower blood glucose response [24]. The mechanisms of action behind RRB are not limited to the starch components. The RRB-induced suppression of postprandial glycemia is likely due to the mechanisms responsible for inhibition of intestinal α-glucosidase [13]. A previous study demonstrated that Riceberry rice extract markedly inhibited α-glucosidase, such as maltase and sucrase [13]. Kinetic analysis also revealed that Riceberry rice extract acts as a mix-type competitive inhibitor against intestinal maltase. Together with the action of phytochemical compounds, the major anthocyanin of Riceberry rice is cyanidin-3-glucoside, which also suppresses the activities of starch digestive enzymes, including α-amylase and α-glucosidase [13,17,25]. The current findings suggest that the postprandial glucose-lowering effect of RRB consumption may also be attributed to its presence of important phytochemical compounds, such as cyanidin-3-glucoside.

Normally, the glycemic index (GI) is a value indicating the immediate effect of dietary carbohydrate-rich foods on the postprandial glucose level after consumption. The results showed that the order of GI was HMB > WB = RRB. The observed results of RRB represented strong support for the greater reduction of postprandial glucose and insulin. Our findings are in agreement with an in vitro study of Thiranusornkij et al. indicating that HMB had a higher predicted GI than RRB [17]. Although WB showed a similar effect on the postprandial glycemic response and GI to RRB, it is not considered as a major source of staple food for people who have a specific condition. Wheat products contribute to negative impacts on human health for those who experience allergies and intolerances, especially in celiac diseases, non-coeliac gluten sensitivity, gluten ataxia, and dermatitis herpetiformis [26]. Therefore, RRB may be a suitable food that is helpful in managing the glycemic response in those people.

During carbohydrate metabolism, postprandial hyperglycemia directly promotes oxidative stress by inducing formation of reactive oxygen species, reducing antioxidant defenses, and increasing oxygen radical-induced protein and lipid peroxidation [27]. To this regard, abnormal changes that occur during hyperglycemia are likely to contribute to microvascular and macrovascular diseases, especially in diabetic patients. Several studies revealed that an increase in postprandial hyperglycemia led to the reduction of antioxidant capacity in blood circulation [28]. Remarkably, consumption of diets containing antioxidants attenuated a reduction of plasma antioxidant capacity in subjects who consumed carbohydrate-rich foods. Moreover, this action consequently suppressed the formation of oxygen radical-induced lipid peroxidation [10,29]. The main finding of our study is that consumption of RRB clearly increased the level of FRAP, indicating that the ability of plasma to scavenge free radicals was improved. In the meantime, the reduction of the FRAP level was seen in subjects who consumed WB and HMB. Interestingly, increasing levels of FRAP has usually been attributed to the consumption of high levels of polyphenolic antioxidants [10]. It was found that Riceberry rice had a higher polyphenol content and FRAP value than Hom Mali rice [17]. Hence, we assumed that anthocyanins in RRB might be responsible for the increase in plasma antioxidant capacity. It has been reported that cyanidin-3-glucoside and peonidin-3-glucoside, the major anthocyanin in RRB, act as a natural antioxidant with high reactivity toward reactive oxygen species (ROS) because of its capability to donate hydrogen atoms or transfer electron from several hydroxyl groups to free radicals and unpaired electrons [30]. However, increases in plasma antioxidant capacity might be accompanied with other bioactive polyphenols in RRB. To confirm this hypothesis, further study is needed to characterize the postprandial concentration of individual polyphenol after ingestion of RRB.

In addition to oxidative damage of proteins, increasing production of ROS induces the oxidation of sulfhydryl groups in thiol-containing molecules [31]. The reduction in postprandial plasma protein thiol can be referred to a marker of oxidative stress in the presence of free radicals. Several studies have shown that protein thiols are decreased in clinical states correlated with increased oxidative stress, including autoimmune diseases [32] and diabetes [33]. In this study, increases in postprandial plasma protein thiol concentration from the baseline were obtained by consumption of WB, RRB, and HMB. It is noteworthy that this action represents an increasing antioxidative defense system in blood circulation. The chemical compounds of breads may also influence the alteration of postprandial protein thiol. Glutathione (GSH) and related thiol compounds, as well as cysteine and cysteinyl-glycine, are naturally occurring substances found in wheat and rice flour [34]. The present results likely provide a clear indication that increases in the plasma thiol group might be attributed to these compounds in wheat and rice flour.

In general, MDA is produced from the production of reactive oxygen species or free radicals, indicating the increased rate of lipid peroxidation and glucose autoxidation. The findings revealed that postprandial plasma MDA level did not increase at all following ingestion of all breads when compared to the baseline. Our report is inconsistent with the earlier finding indicating that an intake of sucrose (50 g) or glucose (37.5 g) induced an increase in postprandial MDA in healthy subjects [10,35]. The lack of effect from consumption of WB, RRB, and HMB on the changes in postprandial MDA from the baseline may have been because of a different type of carbohydrate composition. In addition, Montes-Nieto et al. also suggested that the amount of the lipid composition in bread might account for the dissimilar outcomes of these studies [35].

In general, GLP-1 has been shown to promote satiety and suppress appetite, and thereby reduce food intake. In the current study, ingestion of the three breads had no effect on increasing postprandial plasma GLP-1. In accordance with our results, consumption of bread containing 50 g of carbohydrates led to a slight increase in postprandial GLP-1 than that of wholemeal bread [36]. Gonzalez-Anton et al. explained that the amount of bread (50 g) might be a low isocaloric diet which could not provide the stimulatory effect on the release of GLP-1 mediated induction of satiety [36]. Furthermore, the three breads increased the scores of satiety and fullness with concomitant reduction of hunger and desire to eat 30 min after consumption. However, the subjective rating scores of satiety, fullness, hunger, and desire to eat were similar following WB, HMB, and RRB consumption. Although differences in the total amount of resistant starch and amylose were reported in Riceberry rice flour, Hom Mali rice flour, and wheat flour [17], they were not attributed to postprandial GLP-1 response and the rating score of satiety. Belobrajdic et al. demonstrated that the consumption of wholemeal and refined flour bread did not show different responses on postprandial plasma GLP-1 and the subjective rating score of satiety [37]. Nevertheless, other studies yielded inconsistent results that a diet containing high resistant starch had higher rating scores of hunger and desire to eat than low-amylose products [38,39]. Further studies are necessary to clarify this inconsistency regarding the effect of high and low amylose content in bread on the alteration of gut hormone and satiety.

## 5. Conclusions

A postprandial rise in plasma glucose and insulin elicited by the consumption of anthocyanin-rich Riceberry rice bread was less than that following the consumption of HMB. In addition, an intake of RRB led to a higher increase of FRAP level compared with WB and HMB. However, all breads similarly affected the protein thiol group, MDA level, and appetite ratings. The findings suggest that consumption of RRB was more effective on reduction of postprandial glucose than that of HMB, whereas it had a higher ability to improve the plasma antioxidant status than that of WB. Therefore, anthocyanin-rich Riceberry rice can be used as a natural ingredient for gluten-free bread in order to manage postprandial glucose with a concomitant improvement of antioxidant status in healthy participants. Further study is needed to investigate the long-term effects of RRB with different compositions of macronutrients.

## Figures and Tables

**Table 1:** Composition and nutritional contribution of the test breads.

Proximate Composition (%)	Wheat Bread	Riceberry Rice Bread	Hom Mali Bread
Total Carbohydrate	44.48	42.93	46.98
Total dietary fiber	5.46	3.94	3.76
Available carbohydrate	39.02	38.99	43.22
Protein	10.0	8.16	7.63
Total fat	4.95	5.87	4.7
Moisture	39.0	41.28	39.13
Ash	1.57	1.76	1.49

**Table 2:** Participant characteristics.

	Mean ± SEM
Age (years)	24.29 ± 0.67
BMI (kg/m^2^)	21.29 ± 0.34
Fasting blood glucose (mg/dL)	79.71 ± 3.40
Fasting total cholesterol (mg/dL)	180.48 ± 7.71
Fasting triglyceride (mg/dL)	56.86 ± 4.71
Fasting LDL cholesterol (mg/dL)	121.19 ± 6.22
Creatinine (mg/dL)	0.70 ± 0.02
BUN (mg/dL)	11.33 ± 0.54

All values are means ± SEM, *n* = 16.
